# Obtaining medication histories via telepharmacy: an observational study

**DOI:** 10.1186/s40545-023-00573-w

**Published:** 2023-06-08

**Authors:** Martina Francis, Peter Francis, Asad E. Patanwala, Jonathan Penm

**Affiliations:** 1 Department of Pharmacy, Royal Prince Alfred Hospital, Camperdown, NSW Australia; 2 Faculty of Medicine and Health, School of Pharmacy, The University of Sydney, Camperdown, NSW Australia; 3 Department of Neurology, Blacktown Hospital, Blacktown, NSW Australia; 4 Department of Pharmacy, Prince of Wales Hospital, Randwick, NSW Australia

**Keywords:** Telepharmacy, Medication errors, Best possible medication history, Hospital admission, Transition of care

## Abstract

**Background:**

Medication reconciliation is an effective strategy to reduce medication errors upon hospital admission. The process involves obtaining a best possible medication history (BPMH), which can be both time-consuming and resource-intensive. During the COVID-19 pandemic, telepharmacy was used to reduce the risk of viral transmission. Telepharmacy is the remote provision of pharmacy-led clinical services, such as obtaining BPMHs, using telecommunications. However, the accuracy of telephone-obtained BPMHs has not yet been evaluated. Therefore, the primary aim of this study was to evaluate the proportion of patients who have an accurate BPMH from the telephone-obtained BPMH compared to an in-person obtained BPMH.

**Methods:**

This prospective, observational study took place in a large tertiary hospital. Recruited patients or carers had their BPMH obtained by a pharmacist over the telephone. The same patients or carers then had their BPMH conducted in-person to identify any deviations between the telephone-obtained and in-person obtained BPMH. All telephone-obtained BPMHs were timed with a stopwatch. Any deviations were categorised according to their potential consequence. An accurate BPMH was defined as having no deviations. Descriptive statistics were used to report all quantitative variables. A multivariable logistic regression was conducted to identify risk factors for patients and medications for having medication deviations.

**Results:**

In total, 116 patients were recruited to receive both a telephone-obtained and in-person obtained BPMH. Of these, 91 patients (78%) had an accurate BPMH with no deviations. Of the 1104 medications documented across all the BPMHs, 1064 (96%) had no deviation. Of the 40 (4%) medication deviations, 38 were deemed low-risk (3%) and 2 high-risk (1%). A patient was more likely to have a deviation if they are taking more medications (aOR: 1.11; 95% CI: 1.01–1.22; p < 0.05). A medication was more likely to have a deviation if it was regular non-prescription medication (aOR: 4.82; 95% CI: 2.14–10.82; p < 0.001) or ‘when required’ non-prescription medication (aOR: 3.12; 95% CI: 1.20–8.11; p = 0.02) or a topical medication (aOR: 12.53; 95% CI: 4.34–42.17; p < 0.001).

**Conclusions:**

Telepharmacy represents a reliable and time-efficient alternative to in-person BPMHs.

## Introduction

Medication reconciliation is one of the most effective strategies to reduce medication errors upon hospital admission [1]. Implementing medication reconciliation upon hospital admission can reduce medication errors by 54% [2], with 59% of those errors have potential to cause harm if not rectified [2]. Medication reconciliation refers to the process in which healthcare professionals partner with patients to obtain a *Best Possible Medication History* (BPMH) and verify it using, at least, one other source (for example, a community pharmacy dispensing history) [1, 3–7]. The BPMH is then reconciled with medications prescribed during transitions of care to identify any deviations and rectify them appropriately [1, 3–7].

During the coronavirus disease 2019 (COVID-19) global pandemic, it was clear that transmission can be reduced by limiting physical contact [8], and thus it was necessary for pharmacy services to be delivered using contactless ways e.g., telehealth. Telehealth pharmacy services, or ‘telepharmacy’, is the remote provision of pharmacy-led clinical services using telecommunications [9, 10]. Examples of current telepharmacy services include after-hours pharmacy consultations, medication chart and drug order reviews, patient counselling and clinical monitoring [9, 11]. This suggests that it may potentially be utilised to support medication reconciliation upon hospital admission.

One study evaluated the use of telepharmacy to obtain medication histories [12]. The study explored the use of videoconferencing by pharmacy technicians to obtain medication histories across five sites in the Ascension Texas hospital network. The results reported that pharmacy technicians had 85% medication history accuracy and were more resource- and time-efficient [12]. In the study, BPMH accuracy was determined by a clinical pharmacist who reviewed the patient’s external prescription history, physician notes and reinterviewing the patient, and the purpose was to determine the accuracy between different healthcare professionals [12]. The study evaluated the accuracy of medication histories obtained by pharmacy technicians via videoconferencing and the time taken. The study did not compare their accuracy to a medication history obtained in-person by the pharmacy technician. In comparison, our study aims to evaluate the accuracy of telephone-obtained BPMHs in comparison to in-person obtained BPMHs, as well as time taken, to examine the potential time-efficiency of telepharmacy.

It has been previously reported that the mean time to interview a patient for a BPMH upon hospital admission is 11.4 min in-person [2]. Comparatively, the mean time reported for a telephone-obtained BPMH is 9 min [12]. Telepharmacy may potentially represent a slightly more time-efficient model in obtaining BPMHs. However, to our knowledge this was the only study that examined the efficiency of telepharmacy [12], and thus this study aims to explore this concept further.

The reason for this study is that telepharmacy is a fairly novel concept and despite its potential, there are concerns surrounding its implementation. A telepharmacy review highlighted three concerns of telepharmacy versus in-person care [13]. These concerns include: (1) effective patient counselling (one study reported that pharmacy students performed patient counselling better in-person that via telepharmacy) [13]; (2) operational difficulties (telehealth might be “overwhelming and less spontaneous” [13], as these services require a positive network between various stakeholders) [13]; and (3) reluctance to use technology (with the elderly population being predominantly sceptical of technology use) [13]. Furthermore, a pharmacist may not fully comprehend a patient’s condition via telepharmacy [13]. Without unanimous rules or legislation to govern its implementation, telepharmacy needs to be evaluated for accuracy.

Therefore, the primary aim of this study was to evaluate the proportion of patients who have an accurate BPMH from the telephone-obtained BPMH compared to the in-person obtained BPMH. The secondary objectives were to characterise the number, type, and severity of any deviations; risk-factors associated with those deviations; and the time taken to obtain a BPMH via telephone.

## Methods

### Study design and setting

This was a prospective observational study from September 2021 to November 2022 in a metropolitan, tertiary teaching hospital in the state of New South Wales, Australia.

### Participants

The inclusion criteria were: (1) an adult; (2) admitted to hospital in past 24–48 h; (3) the person responsible for the medications (patient/carer) were cognisant; and (4) the aforementioned person had access to a telephone (either bedside or mobile phone). Patients were excluded if: (1) they were deemed medically inappropriate to be interviewed, e.g., patient had an altered mental status and no available carer to provide information; or (2) there was a communication barrier (patient/carer primarily responsible for the medications could not communicate in English); or (3) the patient was from a community care facility (e.g., nursing home); or (4) had been transferred from a different healthcare facility (e.g., transfer from another hospital); or (5) the patient had their discharge medication reconciliation completed, and were therefore expected to be discharged shortly; or (6) patient had no home medications. Patients were deemed lost to follow-up if a telephone-obtained BPMH was documented, but the patient was unavailable for the in-person obtained BPMH.

### Data collection

For patients who were deemed eligible, the pharmacist would contact either the patient or carer via their bedside telephone or mobile phone. If no response was received, patients or carers were contacted according to the escalation protocol before being excluded from the study (Fig. 3). Once patients or carers were contacted over the telephone, the investigating pharmacist would then obtain a BPMH. The duration of the BPMH was also recorded via stopwatch. At the conclusion of the phone call, the pharmacist notified either the patient that they would come and see them in-person to complete a second medication history check, or they would schedule a time for the carer to come in and have the medication history checked in-person. The pharmacist documented the total duration of the phone consult, including any call backs to the patient/carer. The same pharmacist would then conduct an in-person consultation to verify again the BPMH to identify any deviations. Refer to Appendix A for the methodology flow chart (Fig. 2). The medications were documented as individual generic ingredients, meaning combination medications, were recorded as separate medications. For example, Duodart [dutasteride/tamsulosin] was recorded as two medications, similar to a previous study [14]

The telephone-obtained BPMH and the in-person BPMH were compared for any deviations. An accurate BPMH was defined as having no medication deviations. Medication deviations were defined from a previous study [14], as “any differences between the [telephone]-obtained and pharmacist-obtained BPMH” [14]. These medication deviations were classified according to MedTax (omission, commission, and partial match) [15], and by Anatomical Therapeutic Chemical (ATC) categories [16]. These deviations were then assessed based on their potential consequence, as either insignificant, minor, moderate, major or catastrophic [17]. These consequences are defined as: (i) Insignificant: no harm or injuries, low financial loss; (ii) Minor: minor injuries, minor treatment required, no increased length of stay or re-admission, minor financial loss; (iii) Moderate: major temporary injury, increased length of stay or re-admission, cancellation or delay in planned treatment/procedure. Potential for financial loss; (iv) Major: Major permanent injury, increased length of stay or re-admission, morbidity at discharge, potential for significant financial loss; and (v) Catastrophic: death, large financial loss and/or threat to good will/good name [17]. See Appendix C for examples of medication deviations.

The type and consequence for each deviation was independently categorised by one hospital pharmacist and one hospital medical officer. Any disagreements were discussed with a third senior academic clinical pharmacist. Final decisions were decided by 2/3 consensus. If a patient had more than one medication deviation, the patient was categorised according to the medication deviation with the greatest consequence.

### Sample size

Using a two-tailed one-proportion test to determine an accuracy rate of 80%, similar to face-to-face BPMH [18], alpha of 0.05 and power of 0.95, the minimum sample size required was 110 patients (G*Power version 3.1.9.7).

### Statistical analysis

Descriptive statistics were used to report all quantitative variables. For continuous normally-distributed data, mean (standard deviation) are reported and for categorical data, percentages were reported. Patient demographics (age, gender, emergency, or planned admission, surgical or non-surgical admission, CCI score and total number of medications) were compared between patients who had a medication deviation and those who did not using the Fishers exact test for categorical data and independent t-test for normally distributed continuous data. Analyses was completed at the medication level comparing medications with and without a deviation.

To evaluate the proportion of patients with an accurate BPMH between telephone-obtained and in-person obtained BPMH, a one-proportion z-test was conducted. To identify risk factors for patients and medications for having medication deviations, multivariable logistic regressions was conducted. The dependent variable was if a patient had a medication deviation. Independent variables were age, Charlson Comorbidity Index score, number of medications, type of admission, type of sources used to obtain BPMH. At the medication level, the dependent variable was if a medication had a discrepancy. Independent variables were medication type and route. For all analyses, a p-value of less than 0.05 was considered statistically significant. Statistical analyses were computed using SPSS (version 28, IBM, Chicago, IL).

## Results

### Patient demographics

**Fig. 1:**
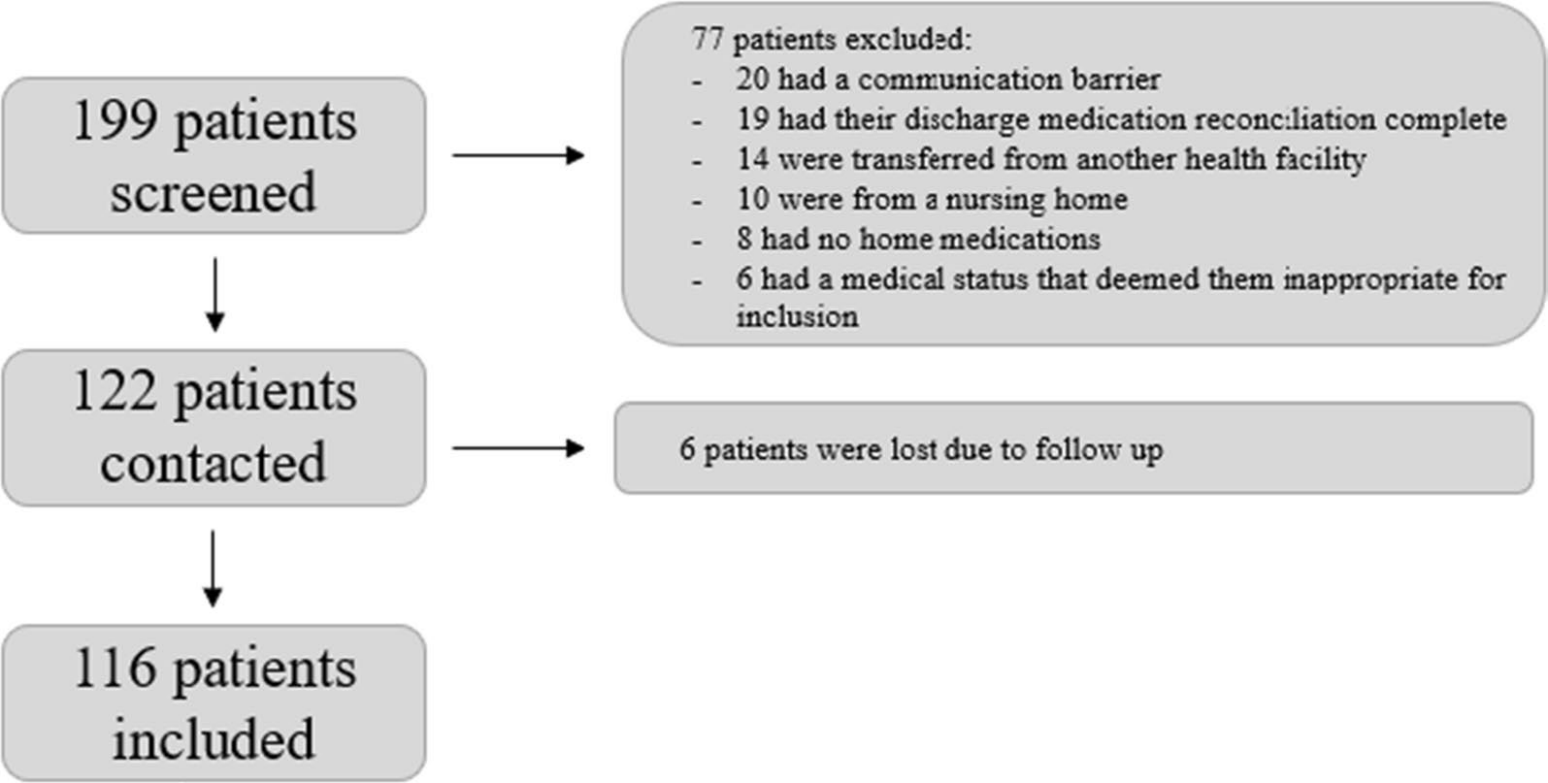
Participant recruitment flowchart

**Fig. 2:**
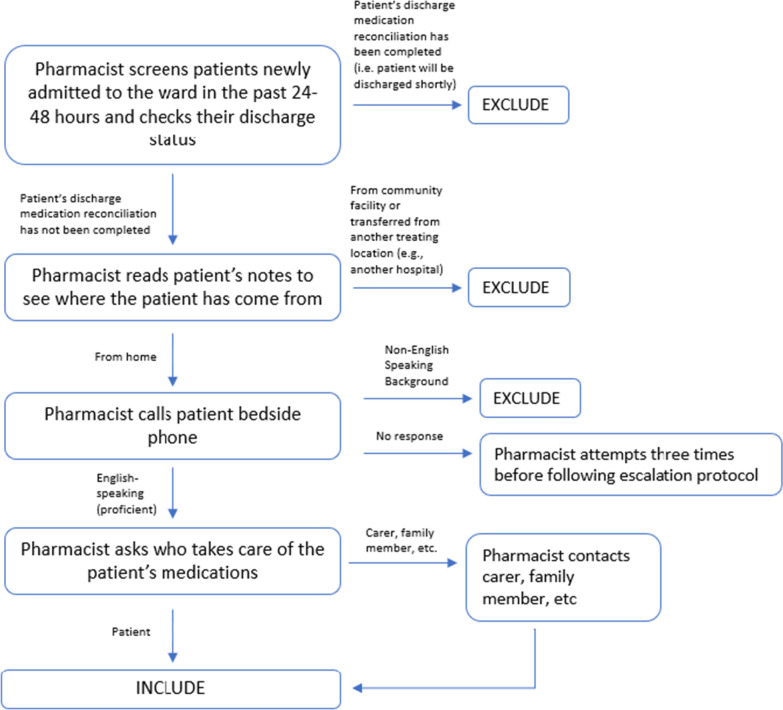
Study flow chart

**Fig. 3:**
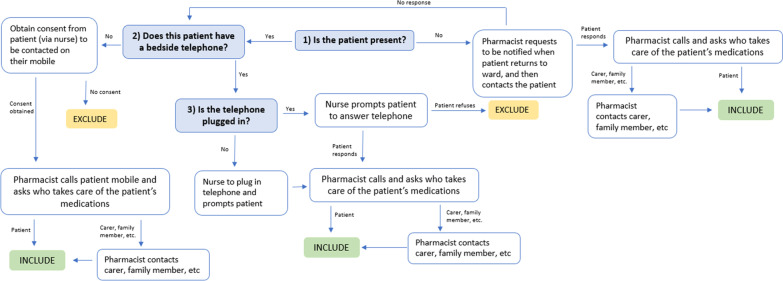
Escalation protocol

From a total of 199 patients screened, 116 were included in the final analysis (Fig. 1). The mean age was 65 (SD = 19) years and 52% were male. Most of the patients’ admission were an emergency (81%) and were non-surgery-related (76%). As an indication of medical-history complexity, the mean CCI score was 4 (SD = 3). On average, patients reported taking 9 (SD = 5) home medications upon admission. This represented an average of 6 (SD = 4) regular prescription medications; 2 (SD = 3) regular non-prescription medications; 0 (SD = 1) when-required prescription medications; and 1 (SD = 1) when-required non-prescription medications. The mean time taken to conduct the patient/carer interview for a BPMH via telepharmacy was 8 min and 25 s (SD = 4 min and 43 s). The difference in patient demographics between those with no deviation and those with a deviation can be found in Table 1.

**Table 1:** Patient demographics (n = 116)

Patient demographics	Deviation risk of best possible medication histories			p-value
	No deviation n = 91 (%)	Deviation n = 25 (%)	Total, n (%)	
Age, n (%)				
< 65 years	38 (41.8)	9 (36.0)	47 (40.5)	0.60
66–75 years	20 (22.0)	8 (32.0)	28 (24.1)	0.30
76–85 years	19 (20.9)	7 (28.0)	26 (22.4)	0.45
> 85 years	14 (15.4)	1 (4.0)	15 (12.9)	0.13
Male, n (%)	49 (53.8)	11 (44.0)	60 (51.7)	0.38
Emergency admission, n (%)	74 (81.3)	20 (80.0)	94 (81.0)	0.88
Surgery-related admission, n (%)	22 (24.2)	6 (24.0)	28 (24.1)	0.99
Charlson Comorbidity Index Score, mean (STD)	4.11 (2.98)	4.32 (2.66)	4.16 (2.91)	0.75
Total number of medicines, mean (STD)	8.80 (5.01)	11.44 (6.44)	9.37 (5.43)	0.03

*STD* standard deviation

### Primary objective—BPMH accuracy

In total, 78% (n = 91/116) of the telephone-obtained BPMHs had no deviations. Of the remaining patients, 11% (n = 13/116) had an insignificant deviation, 10% (n = 11/116) had a minor deviation and 1% (n = 1/116) had a moderate deviation.

There was a total of 1,104 medications documented across all 116 BPMHs. This included 654 (59%) regular prescription medications; 247 (22%) regular non-prescription medications; 158 (14%) ‘when-required’ non-prescription medications; and 45 (4%) ‘when required’ prescription medications. The telephone-obtained BPMHs had 40 deviations (4%, n = 1104) of the medications documented (Table 2). Of the deviations recorded (n = 40), the medication types involved were regular prescription (30%, n = 12/40); regular non-prescription (42.5%, n = 17/40); when-required non-prescription (22.5%, n = 9/40); and when-required prescription (5%, n = 2/40). The MedTax categorisation of these medication deviations was drug omission (67.5%, n = 27/40); drug partially matched (25%, n = 10/40); and drug commission (7.5%, n = 3/40). The risk ratings of the 40 medication deviations were as follows: insignificant (23, 57.5%), minor (15, 37.5%), moderate (2, 5%), major (0, 0%), or catastrophic (0, 0%). Thus, 95% (n = 38/40) were deemed low risk and 5% (n = 2/40) were high risk. These 40 deviations were across 21.6% patients (n = 25/116). Of the 38 low-risk medication deviations, a majority (30%, n = 12/40) were from ‘Alimentary tract and metabolism’ class (8 out of the 12 were due to vitamins/minerals). The 2 high-risk medication deviations were documented for one patient and both medications were regular prescriptions from the ‘Cardiovascular system’ class (100%, n = 2/2).

**Table 2:** Medication deviation details from telephone-obtained best possible medication histories (n = 40/1104)

Medication details	Total, n (%)
Number of medications recorded, n	
Regular prescription medication, n (%)	654 (59.2)
Regular non-prescription medication, n (%)	247 (22.4)
When-required prescription medication, n (%)	45 (4.1)
When-required non-prescription medication, n (%)	158 (14.3)
Total	1104 (100)
Number of medication deviations, n	
Regular prescription medication, n (%)	12 (30)
Regular non-prescription medication, n (%)	17 (42.5)
When-required prescription medication, n (%)	2 (5)
When-required non-prescription medication, n (%)	9 (22.5)
Total	40 (100)
Medication deviation type, n	
Drug omission, n (%)	27 (67.5)
Drug commission, n (%)	3 (7.5)
Drug partial match, n (%)	10 (25)
Total	40 (100)
Anatomical therapeutic chemical classification of medication deviations, n	
Alimentary tract and metabolism, n (%)	12 (30)
Blood and blood forming organs, n (%)	2 (5)
Cardiovascular system, n (%)	3 (7.5)
Dermatologicals, n (%)	4 (10)
Genito urinary system and sex hormones, n (%)	1 (2.5)
Anti-infectives for systemic use, n (%)	3 (7.5)
Nervous system, n (%)	7 (17.5)
Respiratory system, n (%)	6 (15)
Sensory organs, n (%)	2 (5)
Total	40 (100)

Using a one-proportion test, the proportion of patients with a medication deviation was not significantly different between in-person and telephone-obtained BPMHs (p = 0.38).

### Secondary objective—factors associated with patients having an accurate BPMH

In the multivariable logistic regression, patients were more likely to have a medication deviation as total number of medications increased (aOR: 1.11; 95% CI: 1.01–1.22; p < 0.05).

An additional multivariable logistic regression was conducted (Table 3) and showed that a medication is more likely to have a deviation if it is a regular non-prescription medication (aOR: 4.82; 95% CI: 2.14–10.82; p < 0.001) or ‘when required’ non-prescription medication (aOR: 3.12; 95% CI: 1.20–8.11; p = 0.02) compared to regular prescription medicines. In addition, medications were more likely to have a deviation if they were administered topically (aOR: 12.53; 95% CI: 4.34–42.17; p < 0.001) compared to oral medicines.

**Table 3:** Multivariable logistic regression to predict if a patient’s medication was accurately recorded in the telephone-obtained medication history

	Odds ratio (95% CI)	P-value
Medication type		
Regular prescription	Reference	Reference
Regular non-prescription	4.82 (2.14–10.82)	< 0.001
‘When-required’ prescription	2.43 (0.50–11.83)	0.27
‘When-required’ non-prescription	3.12 (1.20–8.11)	0.02
Medication route		
Oral	Reference	Reference
Inhaled or nebulised	0.62 (0.08–4.72)	0.64
Ear or eye	2.04 (0.43–9.61)	0.37
Topical (creams, ointment, patches)	13.53 (4.34–42.17)	< 0.001
Parenteral	2.05 (0.26–16.38)	0.50
Other*	3.21 (0.65–15.81)	0.15

*Other (Sublingual, buccal, per vagina, per rectum, intranasal, irrigation)

## Discussion

The key finding of this study is that telephone-obtained BPMHs appear accurate compared to in-person obtained BPMH. Although medication deviations were identified in this study, the most common deviation types, and classes are consistent with studies that obtained a BPMH a second time in-person. The most common medication deviation type in our study were omissions, with the most commonly omitted medications being non-prescription. This is consistent with other studies that identified omissions [6, 14, 20–25] and vitamins/over-the-counter products [22, 26] as the most common causes for medication deviations when a second in-person obtained BPMH is conducted against an initial in-person BMPH. This suggests that medications which were identified in the second, in-person, encounter, may be due to memory recall bias, as a result of prompting the patient multiple times, and not as a result of telepharmacy. Therefore, telepharmacy represents a viable alternative for pharmacy healthcare professionals to obtain a patient’s BPMH.

Additionally, the mean time for telephone-obtained BPMHs was 8 min and 25 s, which is consistent with the previous telepharmacy study, reporting a mean time of 9 min per BPMH via telephone [12]. This supports the telepharmacy pharmaceutical review that reported a significant increase in the documentation of BPMHs upon admission [27], with the mean time for an in-person obtained BPMH being approximately 11 min and 4 s [2]. This highlights the potential time efficiency of telepharmacy to obtain BPMHs.

This study highlights the potential for policy implementation for telepharmacy in situations where remote pharmacy services are required, or there are staff shortages. Although there are many additional benefits to in-person clinical services, in rural or remote hospitals where there is no or limited pharmacy services, these hospitals can be serviced via telepharmacy. A study examining the delivery of remote pharmacy practices found that multiple hospitals over a large geographical distance can be serviced via telepharmacy [28]. Based on this, hospitals should consider introducing policies promoting telepharmacy practices where face-to-face is not available. Additionally, pharmacy departments where there are staff shortages and, for example, pre-determined BPMH goals are not met, telepharmacy offers a time-efficient alternative. These pharmacy departments may consider introducing a telepharmacy BPMH service as a strategy to improve BPMH percentages. Future studies could explore the feasibility and effectiveness of a remote BPMH service. However, rather than have pharmacists obtain BPMHs, it would be better suited to have pharmacy students and technicians to obtain BPMHs for newly admitted hospital patients. First, obtaining BPMHs requires minimal clinical judgement. Second, studies have proven that pharmacy students and technicians are comparable to pharmacists [12, 14]. Third, having pharmacy students and technicians obtaining BPMHs via telepharmacy represents a cost-effective alternative to pharmacists obtaining BPMHs remotely. Fourth, telepharmacy may represent an opportunity for working remotely, which promotes flexibility in the workforce. Fifth, it supports infection control due to reduced in-person contact. Overall, the role of a hospital pharmacist is supported and allows them to utilise their time for in-person clinical work effectively. Examples of such services include, attending ward rounds, participating in multidisciplinary team decisions, and providing discharge counselling. By having a remote BPMH service, this will allow hospital pharmacists to utilise their clinical skills, in-person, optimally.

### Limitations

First, only one pharmacist was responsible for conducting both the telephone-obtained and in-person BPMH. Although this may have inflated the accuracy of BPMHs, as similar process errors would occur both over telephone and in-person, this method ensures only errors caused by telephone compared to in-person were identified. Second, the potential consequence of a medication deviation was based on subjective assessment. However, a pharmacist and a medical officer completed this task to reduce bias. Third, we did not assess the economic or clinical impact of the identified medication deviations on patient outcomes (e.g., readmission rates). Fourth, telepharmacy eliminates the in-person dimension of patient care, which may compromise on pharmacist-patient rapport. However, this was not assessed in our study and could be a potential qualitative future study. Fifth, this study did not randomise the order of in-person and telephone-obtained BMPHs which raises the possibility of memory recall bias affecting this study.

## Conclusions

Telepharmacy represents an accurate alternative to in-person BPMHs, with a majority of patients having a BPMH with no deviation and almost all medications were documented with no deviation. From the telephone-obtained medication deviations, three-quarters were low-risk; furthermore, the medications were more likely to have a deviation if they are non-prescriptions or topical medications, and therefore telephone-questioning should emphasise these classes of medications. Overall, telepharmacy represents a slightly more time-efficient alternative than in-person BPMHs.

## Data Availability

The data that support the findings of this study are available from Sydney Local Health Distract (SLHD) but restrictions apply to the availability of these data, which were used under license for the current study, and so are not publicly available. Data are however available from the authors upon reasonable request and with permission of SLHD.

## Appendix A

See Fig. 2.

## Appendix B

See Fig. 3.

## Appendix C Examples of medication deviations

**Table:**

Potential consequence of deviation	Example
Insignificant	Drug omission of sorbolene cream
Minor	Omission of day of dulaglutide weekly subcutaneous injection
Moderate	Omission of antihypertensive medication
Major	Commission of buprenorphine topical patch
Catastrophic	Commission of medication to which a patient has an anaphylactic reaction
