# Correction: Obtaining medication histories via telepharmacy: an observational study

**DOI:** 10.1186/s40545-023-00588-3

**Published:** 2023-06-27

**Authors:** Martina Francis, Peter Francis, Asad E. Patanwala, Jonathan Penm

**Affiliations:** 1 Department of Pharmacy, Royal Prince Alfred Hospital, Camperdown, NSW Australia; 2 Faculty of Medicine and Health, School of Pharmacy, The University of Sydney, Camperdown, NSW Australia; 3 Department of Neurology, Blacktown Hospital, Blacktown, NSW Australia; 4 Department of Pharmacy, Prince of Wales Hospital, Randwick, NSW Australia

**Correction: Journal of Pharmaceutical Policy and Practice (2023) 16:69** 10.1186/s40545-023-00573-w

Following publication of the original article [1], it was reported that there was an error in the Funding declaration. The correct Funding declaration is:

Funding

This work was supported by the International Pharmaceutical Federation Hospital Pharmacy Section.

The original article has been updated.
